# Airway dendritic cell maturation in children exposed to air pollution

**DOI:** 10.1371/journal.pone.0232040

**Published:** 2020-05-05

**Authors:** Abigail L. Whitehouse, Naseem Mushtaq, Lisa Miyashita, Benjamin Barratt, Ameerah Khan, Harpal Kalsi, Lee Koh, Michele G. Padovan, Rossa Brugha, Frances R. Balkwill, Andrew J. Stagg, Jonathan Grigg

**Affiliations:** 1 Centre for Genomics and Child Health, Queen Mary University of London, London, United Kingdom; 2 King's College London, London, United Kingdom; 3 Centre of the Cell, Queen Mary University of London, London, United Kingdom; 4 Barts Cancer Institute, Queen Mary University of London, United Kingdom; 5 Centre for Immunobiology, Queen Mary University of London, London, United Kingdom; Telethon Institute for Child Health Research, AUSTRALIA

## Abstract

Urban particulate matter (PM) enhances airway dendritic cell (DC) maturation *in vitro*. However, to date, there are no data on the association between exposure to urban PM and DC maturation *in vivo*. We sought to determine whether exposure of school-age children (8 to 14 y) to PM was associated with expression of CD86, a marker of maturation of airway conventional DCs (cDC). Healthy London school children underwent spirometry and sputum induction. Flow cytometry was used to identify CD86 and CCR7 expression on cDC subsets (CD1c+ cDC2 and CD141+ cDC1). Tertiles of mean annual exposure to PM ≤ 10 microns (PM_10_) at the school address were determined using the London Air Quality Toolkit model. Tertiles of exposure from the 409 children from 19 schools recruited were; lower (23.1 to 25.6 μg/m^3^, n = 138), middle (25.6 to 26.8 μg/m^3^, n = 126), and upper (26.8 to 31.0 μg/m^3^, n = 145). DC expression was assessed in 164/370 (44%) children who completed sputum induction. The proportion (%) of cDC expressing CD86 in the lower exposure tertile (n = 47) was lower compared with the upper exposure tertile (n = 49); (52% (44 to 70%) vs 66% (51 to 82%), *p<*0.05). There was a higher percentage of cDC1 cells in the lower tertile of exposure (6.63% (2.48 to 11.64) vs. 2.63% (0.72 to 7.18), *p<*0.05). Additionally; children in the lower exposure tertile had increased FEV_1_ compared with children in the upper tertile; (median z-score 0.15 (-0.59 to 0.75) vs. -0.21 (-0.86 to 0.48), *p<*0.05. Our data reveal that children attending schools in the highest areas of PM exposure in London exhibit increased numbers of “mature” airway cDCs, as evidenced by their expression of the surface marker CD86. This data is supportive of previous *in vitro* data demonstrating an alteration in the maturation of airway cDCs in response to exposure to pollutants.

## Introduction

Airway conventional dendritic cells (cDC) are professional antigen presenting cells with the capacity to capture inhaled material, migrate to draining lymphoid tissue and activate naïve T cells to initiate adaptive pulmonary immune responses [1]. The nature of the response induced by DC depends in part on their maturation status; mature DC induce a variety of effector T cell responses whereas immature DC induce regulatory responses or T cell anergy. A number of ‘danger signals’ such as cytokines, nucleotides, reactive oxygen intermediates, and neuromediators have the capacity to induce maturation of DC [2]. In addition, there are phenotypically distinct subsets of DC which show some evidence of functional specialisation. For example, cDC1 defined in humans by expression of CD141 (BDCA3), have a high capacity to cross-present antigens to activate CD8+ T cells and preferentially promote T helper type 1 (Th1) responses. In contrast, cDC2, defined by expression of CD1c (BDCA1), efficiently promote the development of a wide range of effector CD4 T cell responses [3].

Many properties of DC, including their maturation status, are determined by local environmental cues. In the airways a putative maturation signal is inhaled particulate matter (PM) air pollution [4]. To date, the role of airway DC in the reported adverse effects of air pollution in children such as suppression of lung function growth [5], increased risk of pneumonia [6] and increased risk of new-onset asthma [7], remains unclear. However, a consistent finding in studies using animal models and human airway cells *in vitro* is that carbonaceous PM and other pollution-related molecules stimulate DC maturation, as indicated by increased expression of the co-stimulatory molecules CD80 and CD86 (required for T cell activation), as well as other maturation-associated molecules including CD83, and CCR7 (required for trafficking to draining lymph nodes) [8–13].

Chan *et al*. for example, reported that short-term exposure of murine bone marrow derived DC to diesel exhaust PM increases expression of CD86 [14], de Haar *et al*. reported increased expression of CD86 and CD80 on DC from lymph nodes in mice after intranasal instillation of ultrafine carbon black P [11], and Ferry *et al*. reported increased expression of CD86 on human monocyte derived DCs incubated with combustion-derived PM [15]. The capacity of pollutants to stimulate DC maturation is not limited to PM since Alexis *et al*. reported increased expression of CD80 in response to either LPS or ozone [16, 17]. Most recently Pfeffer *et al*. reported increased expression of CD83 (an alternative maturation marker) and subsequent enhanced priming of CD8 T lymphocytes by ultrafine PM exposed human DCs [18].

In a previous study in adults we identified airway DC in induced sputum samples using flow cytometry and that DC from patients with asthma are more mature compared with healthy controls, as evidenced by a higher proportion expressing increased CD86 [19]. Subsequently we identified airway DC in the small number of airway cells in induced sputum from school age children with asthma [20]. Since exposure to PM increases DC maturation in vitro, the primary aim of the present study was to test the hypothesis that children exposed to higher annual mean PM_10_ at their school address have increased expression of the maturation markers (CD86, CCR7) in airway DC.

## Methods

### Recruitment

Primary and secondary schools (i.e. schools for children 8 to 14 yr.) within the Greater London (UK) area were contacted. In schools that agreed to participate, written informed consent was provided by parents in addition to children’s verbal assent. An educational workshop on air pollution called “Something in the Air”, devised with the support of staff from Queen Mary University’s outreach science learning centre, Centre of the Cell, was delivered to all children within a class [21]. During this activity, sputum induction was undertaken by up to a maximum of 15 children per class per day. Children were excluded if they had a chronic lung condition such as asthma, or self-reported smoking, or had moved school over the previous year. Parental smoking was self-reported by the children.

### Lung function

Pre- and post-bronchodilator (400 μg salbutamol by metered dose inhaler and large volume spacer) forced expiratory volume in 1 second (FEV_1_), and forced vital capacity (FVC)) was performed according to ERS/ATS guidelines [22] using a portable spirometer (Microlab spirometer, CareFusion). Lung function data were converted to z-scores using the GLI desktop tool which adjusts for ethnicity [23].

### Dendritic cell phenotype

Dendritic cell receptor expression was analysed in induced sputum cells obtained after inhalation of nebulised 3.5% saline for a maximum of 20 min [24]. Sputum was processed within 4 hours of collection as previously described [25]. Briefly, all visible airway plugs were selected and vortexed in the presence of 0.1% dithiothreitol (Sigma-Aldrich, USA) to facilitate mucolysis. Samples were then centrifuged and resuspended in PBS. A small aliquot was cytocentrifuged (Shandon Cytospin 4 Thermo Scientific) for inflammatory cell differential count (%) obtained from 300 leucocytes, as previously described [26].

**Fig 1:**
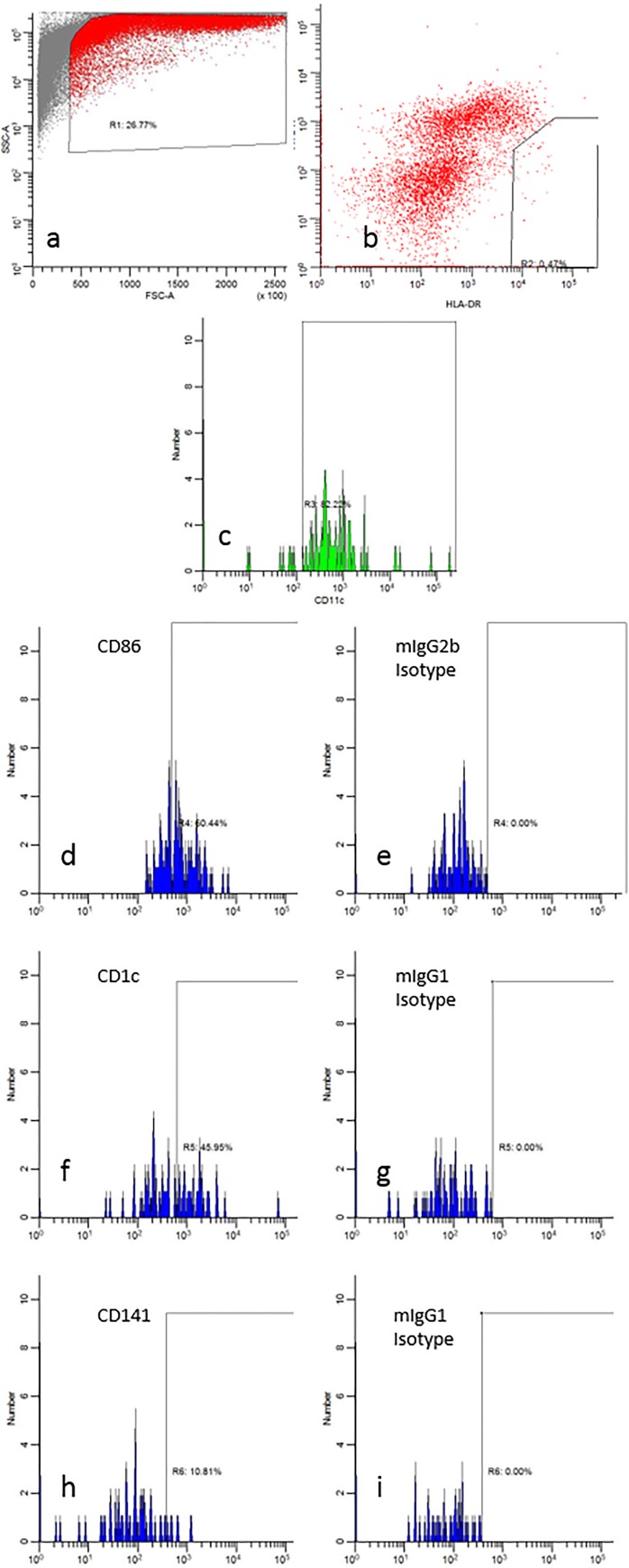
Flow cytometry identification of airway Dendritic Cell (DC) populations. a) Viable sputum cells were identified on the basis of size and granularity (region R1). b) DCs were identified as a HLA-DR+Lineage- population and gated for further analysis (R2). c) CD11c expression was determined relative to the fluorescence levels of autologous non-myeloid sputum cells in the same sample. d-i) Airway DC expression of costimulatory molecule CD86 was determined relative to isotype‐matched control mAbs. Mature DCs defined as HLA-DR+Lin-CD11c+CD86+. f-g) Airway DC expression of subset marker CD1c was determined relative to isotype‐matched control mAbs. cDC2 DCs defined as HLA-DR+Lin-CD11c+CD1c+.h-i) Airway DC expression of subset marker CD141 was determined relative to isotype‐matched control mAbs. cDC1 DCs defined as HLA-DR+Lin-CD11c+CD141+. Percentage values in the figure refer to the results for the individual.

For DC analysis, the processed induced sputum samples were stained with fluorochrome-conjugated monoclonal antibodies and analysed by flow cytometry using our previously reported methods [19, 20]. Briefly, CD11c+ cDCs were identified in the HLA-DR+Lin- population, where Lin is a mixture of antibodies to CD3, CD14, CD16, CD19, CD20 and CD56. (BioLegend, San Diego, USA) which permits the exclusion of T cells, macrophages, neutrophils, B cells and natural killer cells, respectively. cDC subsets were defined by expression of CD141 (cDC1) or CD1c (cDC2) (Miltenyi Biotec, Germany) [27] and maturation status of all cDCs was assessed by staining with anti-CD86 and anti-CCR7 [10] (Fig 1). The gating strategy used was based on the previously validated method used by McCarthy *et al*. [19]. Flow cytometry data were acquired using a BD LSRII flow cytometer with FACSDiva v6.1.03 software (BD Biosciences, USA), and data were analysed using WinList 7.1 software (Verity Software House). If samples had less that 50000 events they were excluded from analysis. Expression of CD1c, CD141, CD86 and CCR7 was determined with reference to staining with contemporaneous isotype-matched control antibodies as shown in Fig 1D–1I. The percentage of CD11c+ cDC expressing each subset or maturation marker were recorded. All samples were analysed in a single sitting for each school group. A viability stain was not routinely included as part of the panel, since exploratory analysis demonstrated that the >90% of the cDC population was viable (data not shown).

### Modelled exposure to air pollution

The London Air Quality Toolkit (LAQT) was used to establish the mean 12-month PM_10_ exposure prior to the date of sputum induction at each school’s post (ZIP) code. The LAQT is a nested dispersion model that produces estimates of pollutant concentrations at a 20m grid resolution using a detailed urban emissions inventory [28].

### Atopy

Atopic status was determined by either child-reported hay fever, eczema, food allergy or history of previous wheeze, or a positive skin prick test done on the day of sputum induction using a multiple head applicator (Multi-Test II, Lincoln Diagnostics, Decatur, IL, USA). Allergens used were; cat hair, grass pollen, tree mix, *Aspergillus fumigatus*, *Dermatophagoides pteronyssinus*, histamine (positive control) and saline (negative control).

### Statistical analysis

Demographic data is summarised as mean ± SEM or mean (SD). All other data are summarised as median (IQR) and compared by Mann Whitney test. The study’s primary outcome was comparison of CD86 expression between lower and upper tertiles. Secondary analysis including the middle tertile shown in S1 Fig. Significance was determined as a *p* value of <0.05. Analyses were performed using GraphPad Prism version 7.1 (GraphPad Software, San Diego, CA, USA). Analyses were performed by an investigator blinded to pollution exposure status.

### Ethical approval

This study was approved by the UK Health Research Authority Research Ethics Committee (reference 13/LO/0440).

## Results

**Fig 2:**
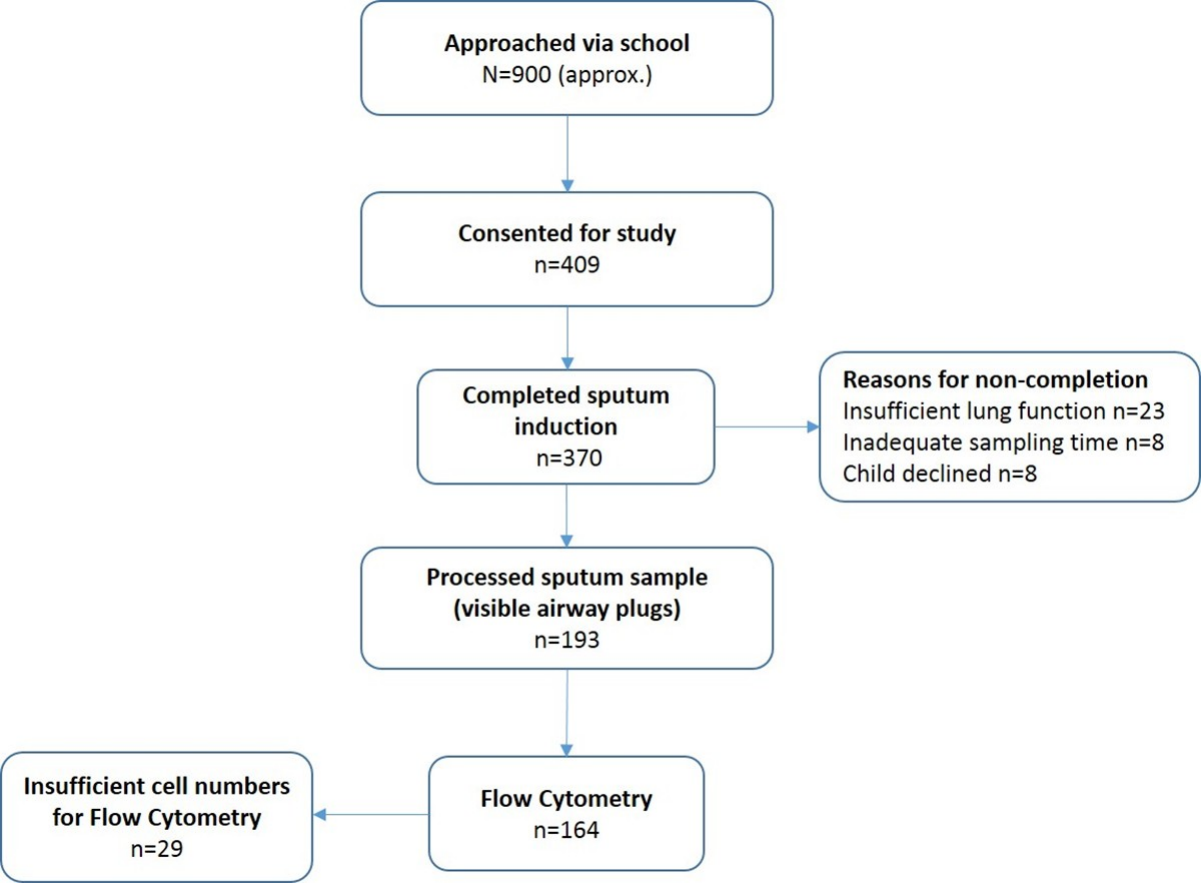
Recruitment to study.

A total of 409 children (mean age; 11.1 ± 0.12 y), were recruited from 19 schools in Greater London between 2013 and 2015, and 370 underwent sputum induction (Fig 2, Table 1). Schools were visited on at least two occasions with up to two school years involved (38 school visits).

**Table 1:** Demographics, atopic status, and induced sputum inflammatory cell differential by tertile of air pollution exposure.

	Tertile of Annual Mean PM_10_ Exposure at School					
	Lower		Middle		Upper	
**n**	138		126		145	
**DC analysis (n)**	47		68		49	
**Age, yr. mean (SEM)**	11.3 (0.07)		11.1 (0.12)		11 (0.13)	
**Male/Female (n) %**	46:92		69:57		89:57	
	33:67		55:45		61:39	
**Height cm, (mean)**	148.1		148		147.6	
**Weight Kg, (mean)**	44.7		44.2		41.9	
**Ethnicity**	n	%	n	%	n	%
**Caucasian**	55	39.9	59	46.8	61	42.1
**African American**	27	19.6	32	25.4	24	16.6
**Asia**	6	4.3	3	2.2	6	4.1
**Indian Subcontinent**	29	21.0	21	16.6	40	27.6
**Mixed**	12	8.7	10	7.9	14	9.7
**Not recorded**	9	6.5	1	0.7		
**Parental Smoking (%)**	30 (21)		36 (26)		31 (21)	
**Atopy**						
**Self-reported hay fever (%)**	18 (13)		23 (18.3)		25 (17.2)	
**Self-reported eczema (%)**	1 (0.7)		1 (0.8)		4 (2.8)	
**Skin-prick testing +ve (%)**	42 (30.4)		35 (27.8)		46 (31.7)	
**SPT +ve for HDM (%)**	22 (16)		19 (15.1)		23 (15.9)	
**SPT +ve for Grass (%)**	13 (9.4)		7 (5.9)		18 (12.4)	
**SPT +ve for Tree (%)**	18 (13)		18 (14.3)		18 (12.4)	
**SPT +ve for Cat (%)**	11 (8)		9 (7.1)		22 (15.2)	
**SPT +ve for Aspergillus (%)**	3 (2.2)		3 (2.4)		5 (3.4)	
**Sputum Leucocyte Differential % (SD)**						
**Macrophages**	85 (10)		73 (17.5)		78 (14.3)	
**Neutrophils**	13 (8.5)		18 (13.9)		16 (10.6)	
**Lymphocytes**	1 (1.2)		7 (10.2)		4.4 (6.7)	
**Eosinophils**	0.7 (1.9)		1 (1.8)		0.4 (0.74)	

Parental smoking defined as any report of parental smoking, in or outside house (self-reported by child).y

Atopy (yes/no) was defined as self-reported atopy symptoms (hay fever, eczema).

SEM (standard error of the mean). HDM (house dust mite), SPT (skin prick test). SD (standard deviation)

Comparative data of participants with DC analysis vs non DC analysis in S1 Table

### Exposure to air pollution at the school

Tertiles for annual mean PM_10_ exposure, calculated from 409 recruited children were; lower tertile; (23.1 to 25.6 μg/m^3^, n = 138), middle tertile (25.6 to 26.8 μg/m^3^, n = 126), and upper tertile (26.8 to 31.0 μg/m^3^, n = 145, cut-off absolute, therefore a participant with a result of 25.6 will be in the lower tertile and 25.61 in the middle tertile). Of note is that the WHO annual mean PM_10_ exposure limit is 20 μg/m^3^ [29]. Demographic data were comparable across exposure tertiles (Table 1), other than gender, where the proportion of boys to girls was 33% versus 66% for the lower tertile and 61% versus 39% for the upper tertile.

### Lung function

**Fig 3:**
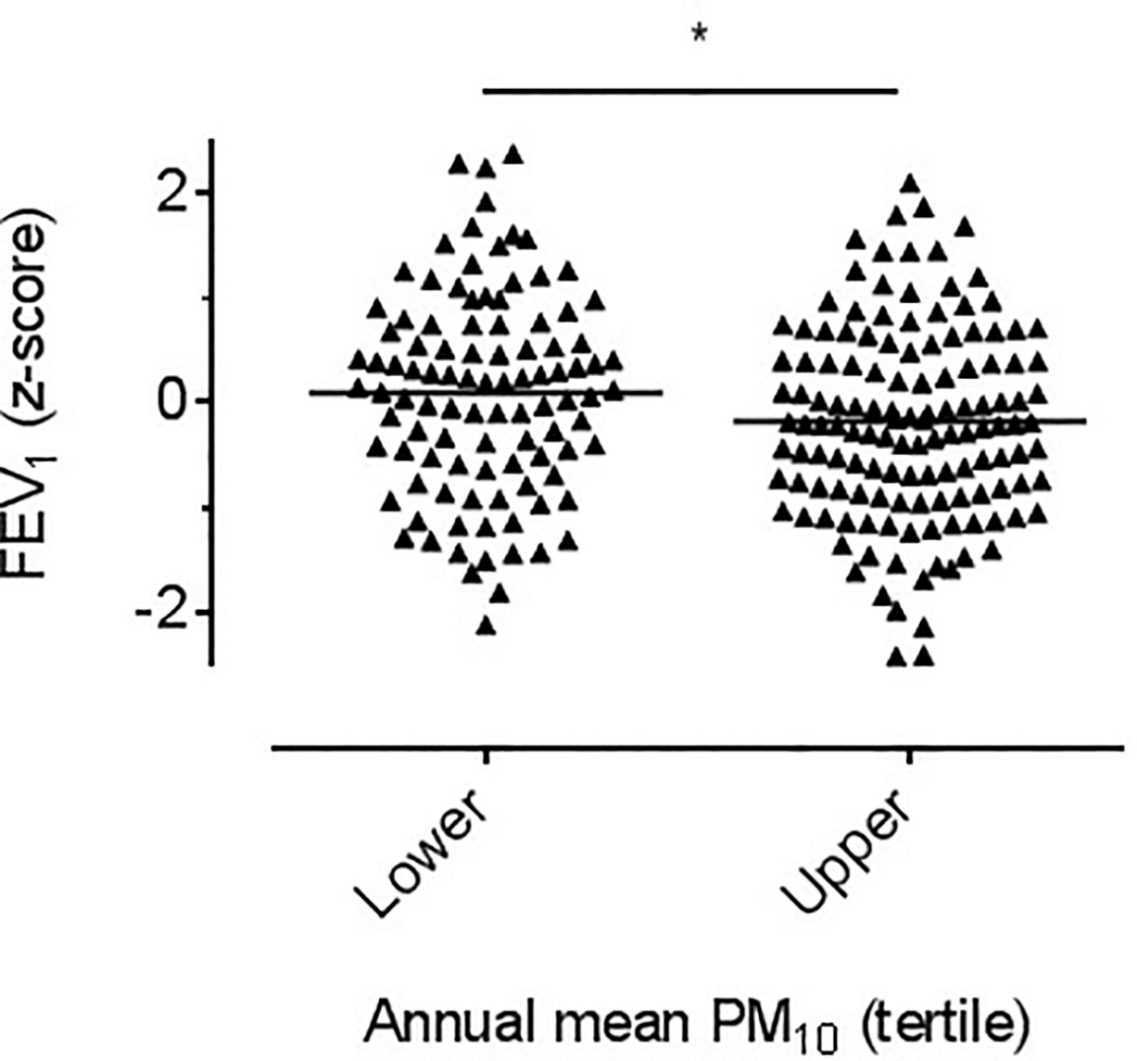
FEV1. Dot plot of FEV1 z-score–each triangle represents an individual participant’s z-score. Bar reflects median. Scaling omits 6 data points. Significant difference between upper and lower tertiles *p<0.05 by Mann Whitney.

Children in the lower exposure tertile had increased post-bronchodilator FEV_1_ compared with children in the upper tertile; (median z-score 0.15 (-0.59 to 0.75) vs. -0.21 (-0.86 to 0.48), *p<*0.05, Fig 3). There was no significant difference for FVC between upper and lower exposure tertiles (median z-score -0.44 (-1.03 to 0.19) vs. -0.26 (-0.91 to 0.29), *p =* 0.27). Post-bronchodilator results were used to avoid the effect of short term influences.

### Airway dendritic cell phenotype

Induced sputum samples from 164/370 (44%) children who underwent sputum induction contained sufficient airway cells for DC phenotyping by flow cytometry. HLA-DR^+^Lin^-^CD11c^+^ cDCs were present in all 164 samples, with n = 47 samples in the lower tertile, n = 68 samples in the middle tertile and n = 49 samples in the upper exposure tertile (Table 1).

### Airway dendritic cell maturation markers

**Fig 4:**
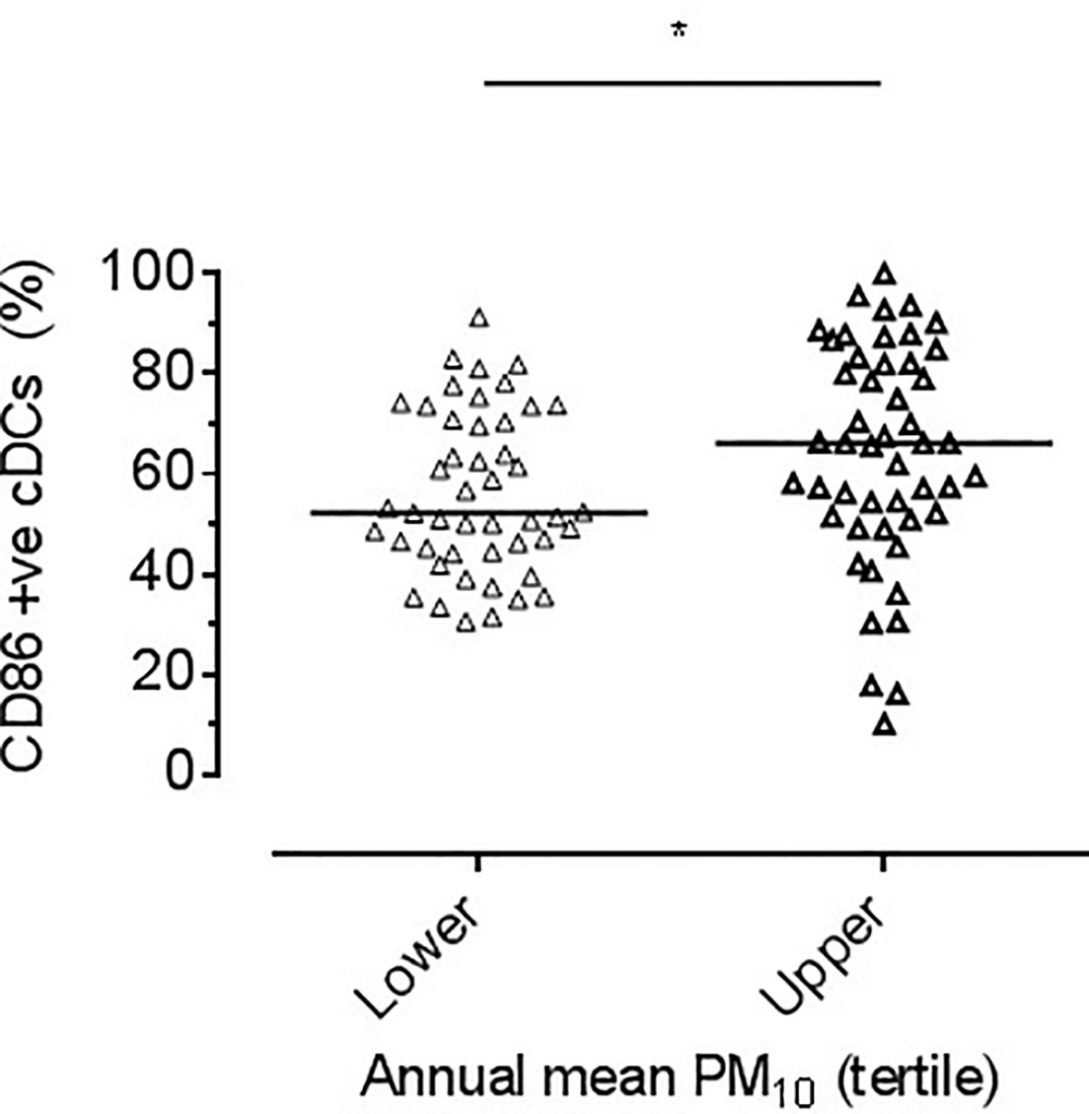
CD86. Dot plot showing the proportion of cDCs expressing CD86 by tertile of mean annual PM_10_ exposure at the school address. Bar reflects median. Significant difference seen between lower and upper tertiles, *P<0.05 by Mann Whitney.

The proportion of cDC expressing CD86 was lower in the lower exposure tertile compared with the upper tertile (median; 52.1% (44 to 70) vs. 66.1% (51 to 82) *p<*0.05, Fig 4). Secondary outcome analysis showed a similar increased proportion between the lower and middle tertile (median; 52.1% (44 to 70) vs.69.7% (55 to 84), *p<*0.001) (S1 Fig). There was no difference in the proportion of DC expressing CCR7 between exposure tertiles (5.0% (2.6 to 8.7) vs.7.8% (4.6 to 12.7), *p =* 0.05). Secondary analysis of middle versus lower also demonstrated no difference in CCR7 expression (*p =* 0.18).

### Dendritic cell subsets

**Fig 5:**
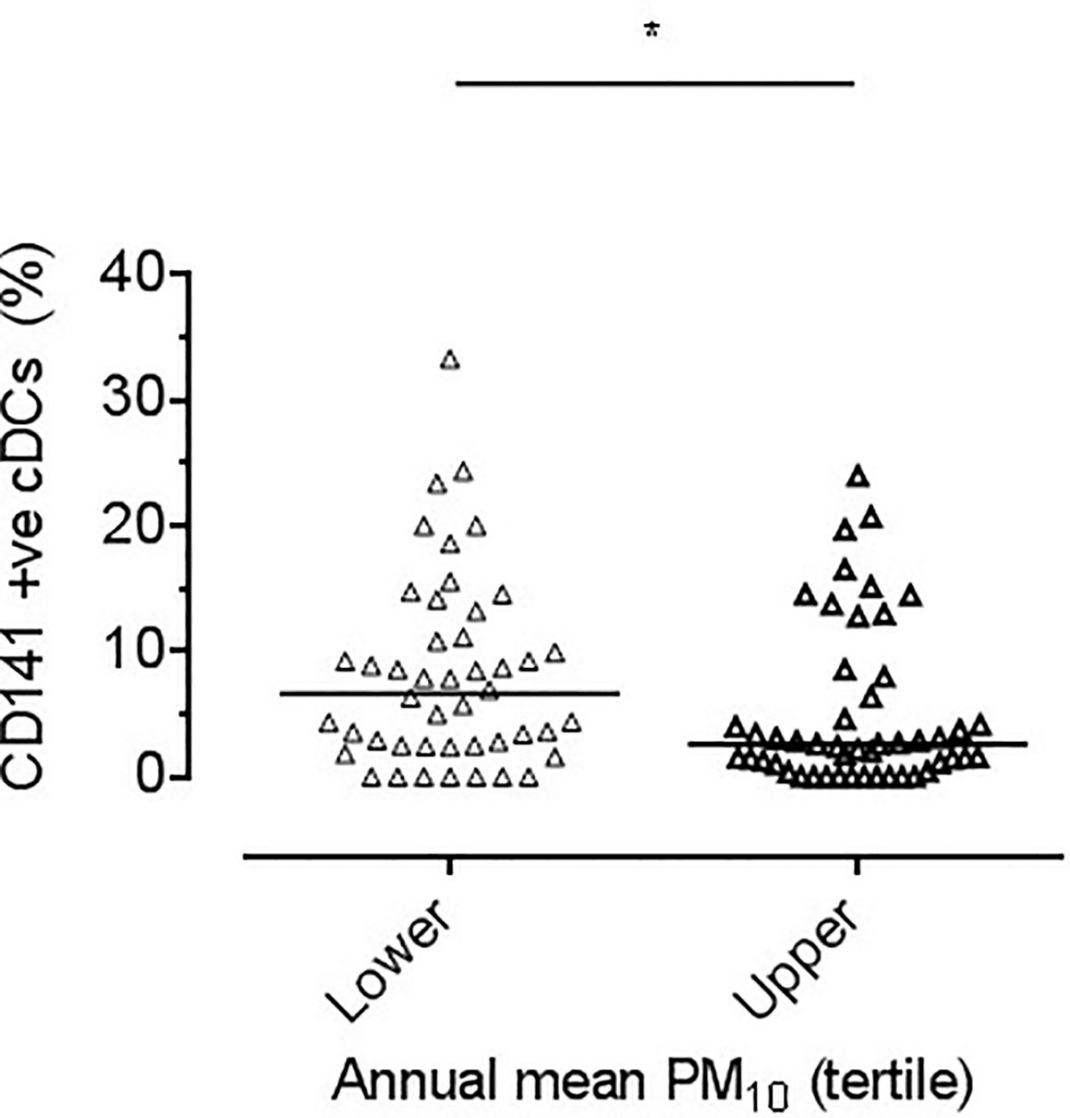
CD1c. Dot plot showing percentage of cDC1 in each individual sputum sample (defined as CD141 positive) divided by PM_10_ tertile. Each triangle represents a single participant’s result. Bar reflects median. Significant difference between lower and upper tertiles, *p<0.01 by Mann Whitney.

There was no difference in the proportion of cDC2 (CD1c positive) cells in sputum from children in the lower exposure tertile and those in the upper tertile (50% (37.1 to 62.2) vs. 43.2% (25.5 to 55.0), *p =* 0.15). However, a secondary analysis showed a lower proportion in the lower tertile compared to the middle tertile (50% (37.1 to 62.2) vs. 54.4% (37.8 to 68.1), *p<*0.01). There was a higher percentage of cDC1 cells in the lower tertile of exposure (6.6% (2.5 to 11.6) vs. 2.6% (0.7 to 7.2), *p<*0.01, Fig 5). Secondary outcome analysis for middle versus lower tertile showed no significant difference (*p =* 0.34).

### Atopic sensitisation and parental smoking

There was no difference in expression of CD86 by sputum cDC between children who reported atopic disease compared with no atopic disease, skin prick positive compared with skin prick negative, and parental smoking compared with no parental smoking (S2 Fig).

### Gender

There was an increased proportion of males in the upper exposure tertile compared to the lower exposure tertile. (61% vs 33%).

Dendritic cell markers were compared by gender. Gender had no significant effect on the subset markers (CD1c *p* = 0.22, CD141 *p* = 0.31), but there was a higher proportion of CD86 positive cells in the male group (66.86% (51.75 to 81.64) vs. 59.19% (45.29 to 73.68), *p*<0.05). There was no effect on CCR7 (*p* = 0.64).

## Discussion

In this study, we sought to assess the effect of exposure to urban PM air pollution on airway cDC subsets and maturation state in children. Specifically, we set out to assess the effect of repeated daily exposure to “usual” levels of air pollution in a high air pollution city, on a highly specialised population of cells that are considered sentinel to airway immune responses. The higher proportion of airway cDC expressing the maturation marker CD86 in children in schools situated in the highest annual PM_10_ areas, is compatible with our hypothesis that increased exposure to PM_10_ is associated with increased DC maturation. These results are compatible with previous studies in murine models. For example, Chan *et al*. reported upregulation of CD86 expression after short term *in-vi*vo exposure of murine bone-marrow derived DC to diesel exhaust particles (DEP) [14], and de Haar *et al*. reported that intranasal instillation of ultrafine particles in human volunteers increased expression of CD86 and CD80 in the lymph nodes [11]. Although a number of cell surface markers including CD86, CD80, and CD40 increase in expression as DC undergo maturation (a process that confers the ability to deliver ‘signal 2’ in order to activate T cells and generate a range of effector responses [3]), we chose to focus on the expression of CD86 since upregulation of this costimulatory molecule is closely linked to competence for T cell activation and the development of airway hyper-responsiveness in an animal model of asthma [30]. However, as a secondary outcome, we also assessed the expression of the chemokine receptor CCR7 required for the lymph node homing response.

Since the number of cells recovered using sputum induction in healthy children was small, we could not assess the functional effect of increased DC maturation. There is, however, evidence to suggest that exposure to PM influences immune pathways associated with disease development. For example, Miyabara *et al*. reported that DEP exposure in conjunction with ovalbumin sensitisation in mice enhanced airway resistance and the production of IgE specific to ovalbumin, suggesting that DEP exposure (and theoretically other pollutants) could exacerbate a pre-existing allergy, or allergic state [31]. Specific evidence of effects of PM on DC is provided by Inoue *et al*. who reported that *in-vitro* exposure of bone-marrow derived DC to DEP resulted in an increased capacity of DC to support T cell proliferation [32], and Bezemer *et al*. reported that *in-vivo* exposure to diesel-enriched PM (DEP), enhances DC activation associated pulmonary inflammation and Th2 responsiveness in mouse lung DC when assessed *ex-vivo* [33]. Additionally, Gao *et al*. reported that upregulation of CD86 on bone-marrow derived DC stimulated by lead was associated with an increased capacity to polarize antigen-specific T cells to Th2 cells [13]. These data suggest that increased DC maturation increases the likelihood that effector T cell responses are generated to otherwise innocuous inhaled antigens and contribute to harmful effects of PM exposure such as asthma exacerbations [6, 34–36]. Our finding of a lower proportion of cDC1 (CD141 positive) cells in children in the upper tertile of exposure was unexpected. Although the functional relevance of changes in cDC1 is unclear, Yu *et al*. reported that CD141 positive blood-derived DCs have a unique capacity to induce IL-4- and IL-13-secreting CD4(+) T cells through OX40 ligand in response to exposure to the live-attenuated influenza virus [37].

There are important limitations to this study. First, it remains unclear to what extent the differences in DC identified in this study, in terms of maturation and subset distribution, reflect modulation of the local DC population or recruitment of distinct DC populations into the tissue, or indeed how DC populations changes over time. There is no published data on variation of DC populations within individuals, but we speculate that both short-term and long-term variation will occur. However it is evident that air pollution does affect CD86 proportions within individual samples. Second, although no evidence of confounding by atopic sensitisation, or exposure to parental cigarette smoke was observed, differences in DC CD86 expression and/or DC subset distribution may reflect exposure to other unmeasured environmental stimuli. Additionally, while the groups had discrepancy in the gender proportions and there was a difference in CD86 proportions across the gender groups, we could find no evidence of known effects of male gender on either dendritic cell phenotype [38], or female gender affecting CD86 expression [39]. We therefore propose that while we cannot rule out gender as an influence it is likely this effect is due to the unequal distribution of the groups. Furthermore there were a significant number of children that did not successfully provide sputum samples for analysis (66%). A comparison of the two groups is provided (S1 Table). There was no significant difference between the two groups in markers of health (Atopy, Lung function, smoking exposure). We did again find a higher proportion of boys in the successful induction group suggesting a possibility that they were more likely to provide a suitable sample and may go someway to explaining the gender discrepancy across tertiles. However the groups were otherwise generally similar and representative of the cohort as a whole. Third, we used modelled exposure at the school and not, as previous studies have done, the home address [40]. We chose to model exposure of the school since: i) children in London may move home but continue to attend the same school, ii) dispersion modelling is problematic for children living in high-rise social housing [41], and iii) effects of air pollution have previously been reported for modelled long-term exposure at the school–for example Chen *et al*. reported lower lung function in children attending a highly polluted primary school in China compared with a low-exposure school [42]. In the present study, indirect evidence that children are indeed exposed to higher levels of air pollution is provided by the small but significant reduction in FEV_1_ in children in the upper exposure tertile, a finding consistent both with Chen *et al*. [42], and other previous studies [26]^,^[43]. There was no association between lung function and the DC maturity markers in this study, likely due to the sample size. Additional evidence of the validity of using the school address to model exposure is provided by a report by UNICEF in children that indicated that nearly 60% of 24 h exposure to BC in a small sample of London school children was from travelling to and from school and being at school [44]. A further study limitation is that the majority of recruited children were exposed to high levels of pollution by World Health Organization (WHO) standards [29]. Indeed, we speculate that if the study had been done in a city where a greater proportion of children lived in areas with cleaner air, the association between exposure and DC CD86 expression would be more pronounced. However, the higher exposures of the majority of children in our study suggests that even modest decrements in air pollution resulting from, for example, the proposed London ultralow emission zone [45], may have effects on the airway immune milieu. In the present study, we found no difference between CD86 expression between the upper and middle tertile data compatible with a French study suggesting that the relationship between exposure to PM and asthma exacerbations is non-linear [46]. Finally, the very low number of cells (children’s sputum samples yielded around 10% of DCs compared with adults) markedly limited the number of markers that we were able to assess. Increased recognition of further heterogeneity amongst DC, as described by Collin *et al*. and Patel *et al*. may allow for improved analysis and a greater understanding of the functional effects of pollution exposure in future studies [3, 47]. The decision to focus on CD86 was made based on previous in-vitro results, however it would be valuable in the future to include other DC maturation markers and other DC markers indicative of other aspects of DC function.

## Conclusions

In conclusion, we found that school children in the highest polluted areas had an increased proportion of mature airway dendritic cells and changes in DC subsets, which provides *in vivo* evidence to support the hypothesis that some of the deleterious effects of inhaled air pollution are driven by immune dysregulation from PM impacting on the lower airways.

## Supporting information

S1 Fig (TIF)

S2 Fig (TIF)

S1 Table (DOCX)

S1 File (XLSX)
