# Supplementary figures and images for: Airway dendritic cell maturation in children exposed to air pollution
Source: PLoS One. 2020 May 5;15(5):e0232040. doi: 10.1371/journal.pone.0232040 (PMC7200006; doi:10.1371/journal.pone.0232040)

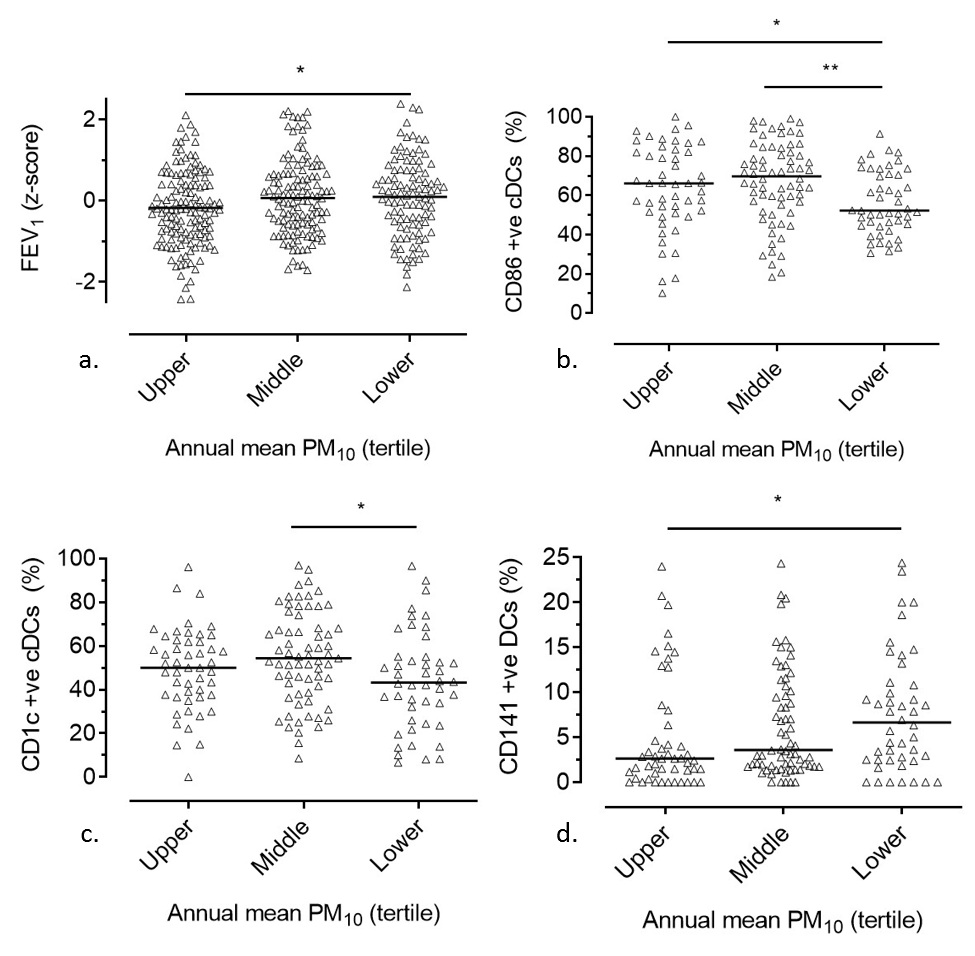

Supplement: S1 Fig — (TIF) [file pone.0232040.s001.tif]

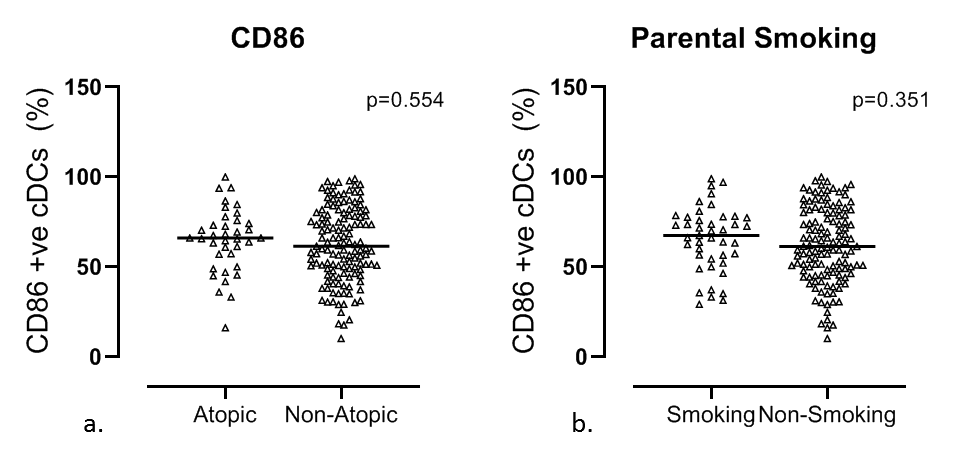

Supplement: S2 Fig — (TIF) [file pone.0232040.s002.tif]
