# Supplementary material for: Airway dendritic cell maturation in children exposed to air pollution
Source: PLoS One. 2020 May 5;15(5):e0232040. doi: 10.1371/journal.pone.0232040 (PMC7200006; doi:10.1371/journal.pone.0232040)
Supplement: S1 Table — (DOCX) [file pone.0232040.s003.docx]

|  | | Sputum Induction | | | |
| --- | --- | --- | --- | --- | --- |
|  | Successful | | Unsuccessful | |  |
| n | 164 | | 245 | |  |
| Age, yr. mean (SEM) | 11.33 (1.22) | | 11.02 (1.22 | |  |
| Male/Female (n)  % | 96:68  58.5:41.5 | | 108:137  44.1:55.9 | |  |
| Height cm, (mean) | 149.1 | | 147.03 | |  |
| Weight Kg, (mean) | 45.22 | | 42.43 | |  |
| PM_10_ median (IQR) | 25.9 (25.5-27.7) | | 25.9 (25.6-26.9) | |  |
| Ethnicity | n | % | n | % |  |
| Caucasian | 74 | 45.1 | 101 | 41.2 |  |
| African American | 45 | 27.4 | 38 | 15.5 |  |
| Asia | 5 | 3 | 10 | 4.1 |  |
| Indian Subcontinent | 25 | 15.2 | 65 | 26.5 |  |
| Mixed | 15 | 9.1 | 21 | 8.6 |  |
| Not recorded | 0 | 0 | 10 | 4.1 |  |
| Parental Smoking (%) | 41 (25) | | 56 (22.9) | |  |
| Atopy | | | | | |
| Self-reported hay fever (%) | 20 (12.2) | | 46 (18.8) | |  |
| Self-reported eczema (%) | 1 (0.6) | | 5 (2) | |  |
| Skin-prick testing +ve (%) | 50 (30.5) | | 73 (29.8) | |  |
| Lung Function | | | | |  |
| FEV_1_ z-score mean (SD) | 0.85 (0.99) | | -0.91 (1.03) | |  |
| FVC z-score mean (SD) | -0.11 (0.99) | | -0.36 (1.04) | |  |
